# The role of uromodulin in cardiovascular disease: a review

**DOI:** 10.3389/fcvm.2024.1417593

**Published:** 2024-07-09

**Authors:** Chengqian Chen, Wentao Zhong, Hao Zheng, Gaoying Dai, Wei Zhao, Yushi Wang, Qi Dong, Botao Shen

**Affiliations:** ^1^ Department of Cardiology Center, The First Hospital of Jilin University, Changchun, China; ^2^ Department of Endocrinology and Metabolism, The First Hospital of Jilin University, Changchun, China

**Keywords:** uromodulin, hypertension, coronary heart disease, kidney, biomarker

## Abstract

Uromodulin, also referred to as Tamm Horsfall protein (THP), is a renal protein exclusively synthesized by the kidneys and represents the predominant urinary protein under normal physiological conditions. It assumes a pivotal role within the renal system, contributing not only to ion transport and immune modulation but also serving as a critical factor in the prevention of urinary tract infections and kidney stone formation. Emerging evidence indicates that uromodulin may serve as a potential biomarker extending beyond renal function. Recent clinical investigations and Mendelian randomization studies have unveiled a discernible association between urinary regulatory protein levels and cardiovascular events and mortality. This review primarily delineates the intricate relationship between uromodulin and cardiovascular disease, elucidates its predictive utility as a novel biomarker for cardiovascular events, and delves into its involvement in various physiological and pathophysiological facets of the cardiovascular system, incorporating recent advancements in corresponding genetics.

## 1 Introduction

In 1873, Italian physician Carlo Rovida made the initial observation of a protein present in human urine, forming tubular patterns, later identified as uromodulin(UMOD), also referred to as Tamm-Horsfall protein (THP). This protein is the most abundantly secreted protein in the urine of healthy individuals and is primarily synthesized by the epithelial cells lining the renal tubules. Initially termed “cilindrina” (1), it wasn't until 1950 that Igor Tamman and Frank Horsfall recognized its potential as a mucin in preventing viral blood clotting, leading to the adoption of the name “Tamm-Horsfall protein” (2, 3). Subsequent research by Muchmore and Decker in 1985 revealed its immunosuppressive properties *in vitro* against T cell proliferation and monocytotoxicity, prompting its renaming as uromodulin (4, 5). Further genomic analysis by Pennica et al. confirmed the equivalence of Tamm Horsfall protein and uromodulin (6). The elucidation of uromodulin's physiology, structure, function, regulation, genomics, and potential clinical applications has gradually unfolded over the past two decades, shedding light on its once-mysterious nature. Extensive research has deepened our understanding of uromodulin's roles in various disease states. While much attention has been paid to its expression as a biomarker for kidney disease, recent years have witnessed a growing body of evidence linking uromodulin to cardiovascular events and mortality through numerous clinical and Mendelian randomization studies. This linkage is comprehensible given the recognized association between chronic kidney disease (CKD) and cardiovascular events (7), alongside the established role of uromodulin in salt-sensitive hypertension (8). Considering hypertension's status as a significant risk factor for various cardiovascular events (9), uromodulin emerges as a potential biomarker extending beyond renal function. This review aims to provide an overview of the current understanding of urinary regulatory proteins, highlighting their newfound implications in cardiovascular diseases such as hypertension and coronary heart disease. It underscores that urinary regulatory proteins serve not only as biomarkers for kidney diseases like CKD but also possess predictive value for cardiovascular events.

## 2 Overview of the structure and biochemistry of uromodulin

**Figure 1:**
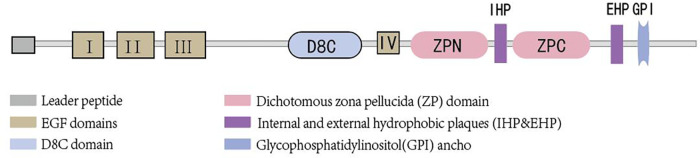
Brief structure of uromodulin.

Uromodulin stands as the most prevalent protein in the urine of healthy adults and is exclusively synthesized by epithelial cells lining the renal tubules. Approximately 90% of uromodulin production originates from cells in the thick ascending limb (TAL), with another roughly 10% synthesized by epithelial cells in the early segment of the distal convoluted tubule (DCT). Its normal daily secretion ranges from 50 to 150 mg in the urine (10, 11).Uromodulin is composed of 640 amino acids (12), possesses a half-life of 16 h (13), and boasts a molecular weight of approximately 80–90 kDa (14, 15). Glycosylation, accounting for nearly 30% of its molecular weight, occurs at eight sites (16). The presence of sialic acid residues renders the protein highly acidic (17), with an isoelectric point of about 3.5 (18). Structurally, uromodulin encompasses multiple domains, including four epidermal growth factor (EGF)-like domains, a cysteine-rich domain (D8C) of unclear function, an internal hydrophobic plaque (IHP), and a dichotomous zona pellucida (ZP) domain facilitating protein polymerization (19) (As shown in Figure 1). The zona pellucida is followed by the external hydrophobic sheet (EHP) and the glycophosphatidylinositol(GPI) ancho. Uromodulin undergoes extensive intracellular post-translational modifications, including N-glycosylation of seven of the eight conserved sites (16) formation of 24 disulfide bridges, and cleavage of the serine protease hepsin at the C-terminus (20), with the endoplasmic reticulum playing an important role in uromodulin processing. Uromodulin was further elucidated using cryo-electron microscopy (cryo-EM) (21). The uromodulin polymerises into filaments with a core formed by a unique interlocking structure of the ZP-N and ZP-C structural domains arranged in a helical pattern with a rise of ∼65 Å and a twist of ∼180° (22, 23). The ZP-N and ZP-C domains have an immunoglobulin-like structure and interact with the ZP linker region by forming a β-fold. After Hepsin cleavage and EHP dissociation, uromodulin monomers are incorporated into growing filaments in a head-to-tail fashion as the activated ZP-C terminus interacts with the ZP-N structural domain of the afferent subunit (23). Although the predominant form of urinary uromodulin (uUMOD) is polymerized, new data suggest that non-polymerized forms exist and that retained EHP lacks GPI anchors (24). In addition to urine, non-aggregated forms of basolateral release also result in detectable serum uromodulin (sUMOD), albeit at significantly lower levels than urine concentrations (25).

## 3 Overview of the physiological functions of uromodulin

Uromodulin serves various physiological functions, encompassing the prevention of urinary tract infections, kidney stone formation, involvement in renal ion transport, and immune regulation, among others (12). Scanning electron microscopy of urine-purified uromodulin reveals a three-dimensional mesh composed of filaments with pore sizes ranging from 0.1 to 1 μm, forming a “fishing net” capable of trapping microorganisms for elimination through urination (26). Imaging studies of uromodulin-urinary pathogen interactions, both *in vitro* and in patient urine samples, demonstrate that uromodulin filaments associate with uropathogens, facilitating bacterial aggregation and potentially impeding adherence and clearance by urination (27). Numerous animal experiments and clinical investigations corroborate the positive correlation between uromodulin levels and protection against urinary tract infections (28–30). The protective effect of uromodulin against renal stones is believed to occur indirectly by inhibiting fiducial protein-mediated endocytosis and up-regulating the activity of TRPV5 channels to enhance calcium reabsorption in the distal convoluted tubule (DCT) (31–33), thereby reducing luminal calcium concentration. However, the precise mechanism and stages of uromodulin's action in crystal nucleation, growth, and aggregation remain to be fully elucidated. Uromodulin also plays a role in renal ion transport, particularly sodium, calcium, and magnesium. Its modulation of magnesium reabsorption involves regulating the cell surface abundance of the magnesium channel TRPM6 at the apical membrane of DCT cells (34, 35). Additionally, uromodulin exhibits potent immunomodulatory properties, activating various inflammatory cells such as neutrophils (36–38), macrophages (39, 40), and dendritic cells (41, 42). Moreover, it serves as a binding ligand for multiple molecules including serum albumin, immunoglobulin G light chains, complement components C1 and C1q, interleukins (IL)-1β, IL-6, IL-8, tumor necrosis factor (TNF)-α, and interferon-γ, via its carbohydrate side chains, thereby contributing to circulatory and renal immune homeostasis (15). Uromodulins exert distinct immune functions across different diseases and environments. Differences in the structure and regulation of uUMOD and sUMOD play important roles in physiological functions in the kidney, urinary tract, and systemic circulation. A growing number of animal experiments and clinical studies have shown that uUMOD levels are associated with an increased risk of salt-sensitive hypertension, while sUMOD appears to have a protective effect on the vascular system by inhibiting vascular calcification, both of which will be focused on later in this review. As the physiological functions of uromodulins are gradually being explored and recognized, they are increasingly recognized as a novel biological marker suggestive of cardiovascular disease.

## 4 Relationship between uromodulin and various cardiovascular diseases

**Figure 2:**
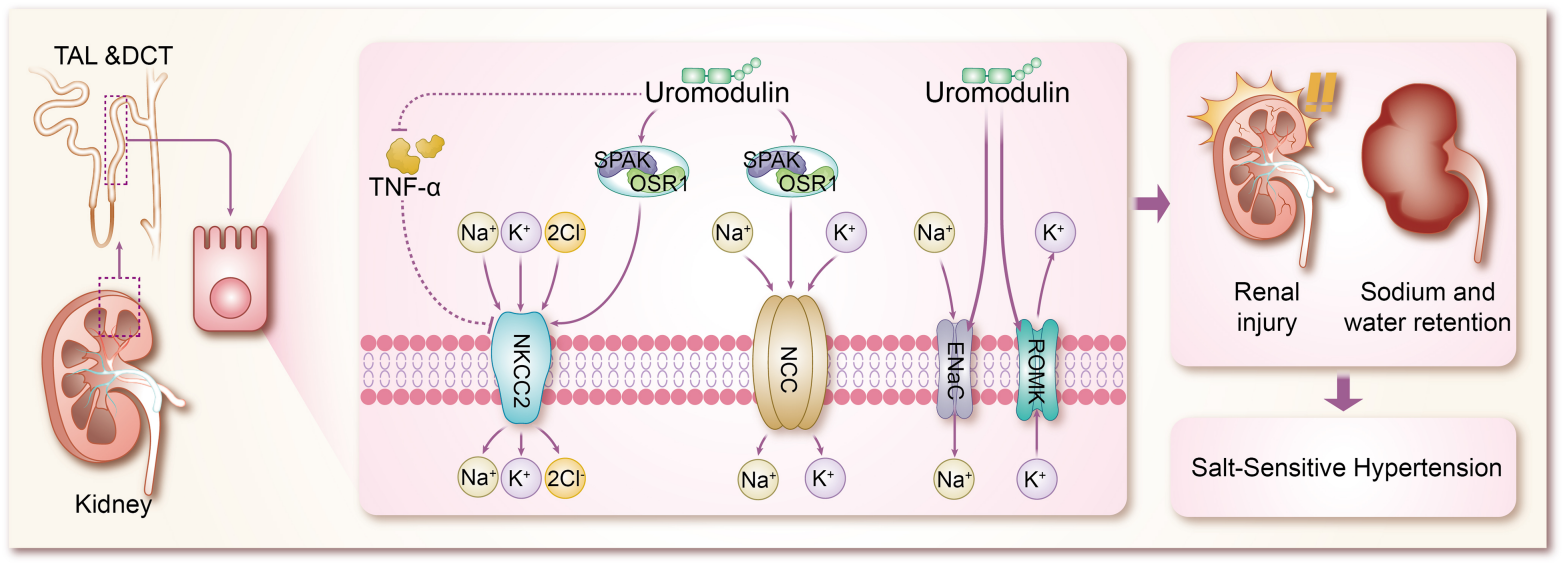
Mechanisms by which uromodulin affects salt-sensitive hypertension. TAL, thick ascending limb; DCT, distal convoluted tubule; TNF-α, tumor necrosis factor-α; SPAK, SPS1-associated proline/alanine-rich kinase; OSR1, oxidative stress-responsive kinase 1; NKCC2, Na + K + -2CI-cotransporter protein; NCC, Na + -CI-cotransporter protein; ENaC, epithelial sodium channel; ROMK, renal outer medullary potassium channel.

**Figure 3:**
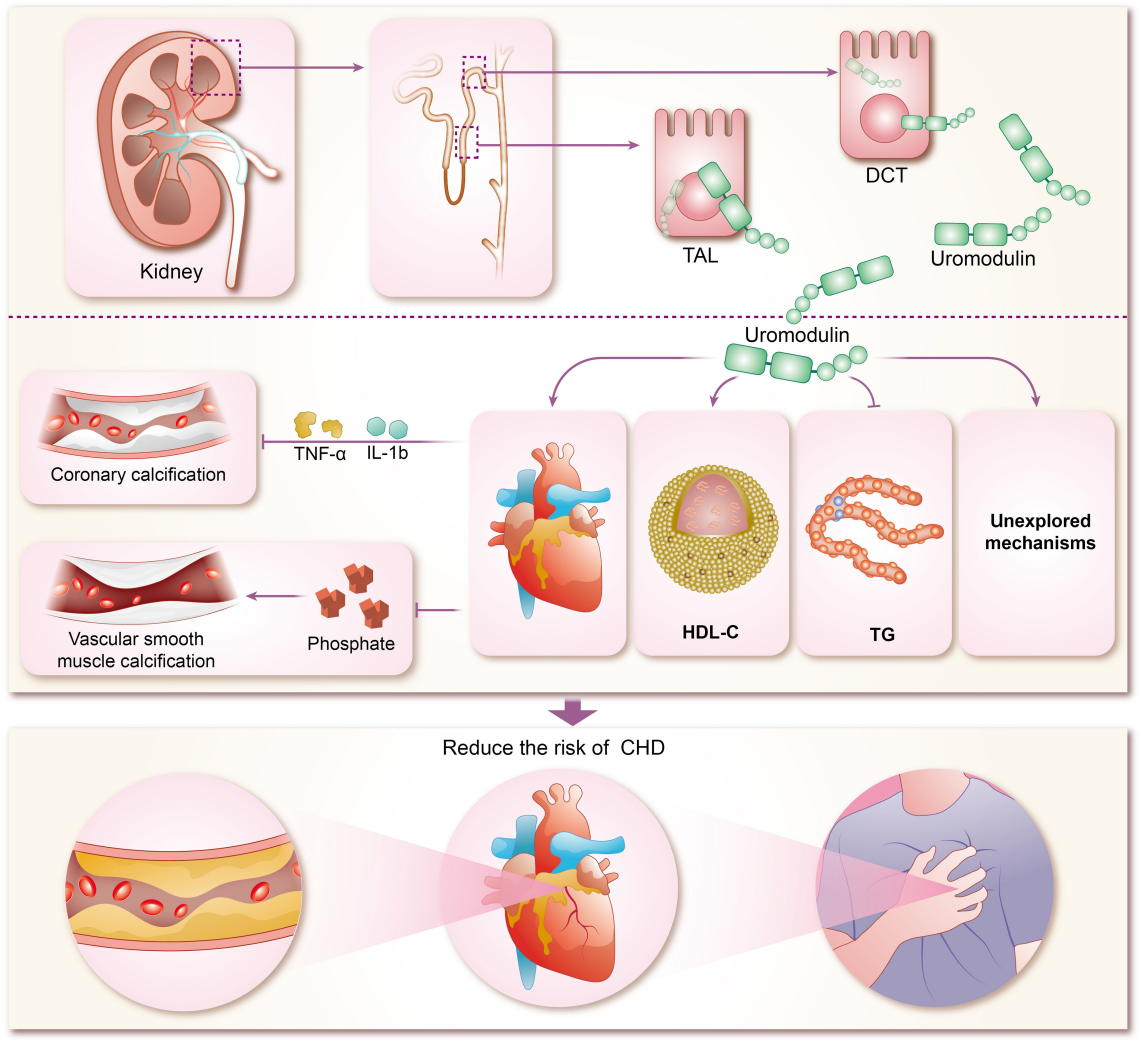
Mechanisms of coronary heart disease risk reduction by uromodulin. TAL, thick ascending limb; DCT, distal convoluted tubule; HDL-C, high-density lipoprotein cholesterol; TG, triglycerides; IL-1b, interleukins-1b; TNF-α, tumor necrosis factor-α; CHD, coronary heart disease.

The mechanisms by which uromodulin affects hypertension and coronary heart disease are shown in Figures 2, 3.

### 4.1 Uromodulin and hypertension

Hypertension, a multifaceted chronic clinical syndrome, stands as a leading cause of various cardiovascular diseases including coronary heart disease and stroke (43). Globally, over a quarter of the population is estimated to suffer from hypertension, totaling more than 1 billion individuals. Its prevalence has been on the rise over the past decade, contributing significantly to the global disease burden and accounting for up to 10 million deaths worldwide (44). Hypertension manifests through numerous variable risk factors (43, 45), such as low potassium intake (46), high sodium intake (46), overweight and obesity (47), unhealthy diet (48), lack of physical activity (49), smoking and alcohol consumption (50), and other factors. However, the pathogenesis of hypertension is complex and is not fully understood. Sodium (Na+) homeostasis plays an important role in the regulation of blood pressure, and small changes in its rate of reabsorption may lead to significant changes in Na + excretion, leading to disturbances in Na + homeostasis and extracellular fluid volume, and ultimately to hypertension (51, 52). In a trial based on testing the effects of sodium on blood pressure modulated by uUMOD levels in the general population, it was found that higher sodium intake was associated with higher blood pressure in a stratified group of people with high levels of uUMOD, whereas subjects with low expression of uUMOD did not show a correlation between blood pressure and sodium intake, which confirms the role of uUMOD in the salt-sensitive component of blood pressure regulation (53). Under basal conditions, systolic blood pressure was significantly lower in UMOD knockout mice compared to wild-type (WT) mice (54). In addition in transgenic mice overexpressing UMOD, treatment with furosemide, a labeled diuretic, significantly enhanced urinary sodium excretion and reduced blood pressure levels (55). These studies using knockout and transgenic UMOD mouse models suggest that umod plays an important role in the development of salt-sensitive hypertension, resulting primarily from sodium reabsorption via Na + K + -2Cl- cotransporter protein (NKCC2) and Na + -Cl- cotransporter protein (54–56). The mechanism by which UMOD has been shown to affect blood pressure is mainly the combination of TAL and DCT to modulate the activity of ion transport proteins in epithelial cells, including the renal outer medullary potassium channel (ROMK) (57, 58), epithelial sodium channel (ENaC) (59), NKCC2 (60), and NCC (8, 61, 62), of which NKCC2 and NCC are the major transport proteins responsible for sodium reabsorption (63). Mutations in NKCC2 and malfunctions in its regulators are known to cause Bartter syndrome, a salt-depleting hypotensive disorder with reduced levels of UMOD, in addition to a significant reduction in NKCC2 phosphorylation in Umod(-/-) mice, where NKCC2 expression is not as strong as in WT mice, and conversely, mice with overexpressed UMOD exhibit salt-sensitive hypertension (64–66). Earlier studies confirmed that uromodulin promotes the activation of NKCC2 in a chloride-sensitive manner (66), but did not identify a specific mechanistic process for its activation, and similar to NKCC2, uromodulin also activates NCC in the DCT (67). It was subsequently demonstrated in a growing number of animal studies that uromodulin induces a significant increase in NKCC2 and NCC phosphorylation and its activity, leading to the up-regulation of NKCC2 and NCC through the regulation of the SPS1-associated proline/alanine-rich kinase/oxidative stress-responsive kinase 1 (SPAK-OSR1) (55, 66–70), which are involved in the pathogenesis of salt-sensitive hypertension (71–75). In previous studies of salt-sensitive hypertension, it has been shown that stimulation of SPAK/OSR1 activates renal ion channels including NKCC2, NCC, ENaC, and ROMK (76), so it is likely to be hypothesized that UMOD also promotes the expression of ROMK and ENaC through activation of SPAK-OSR1. The role of ENaC in salt-sensitive hypertension is self-evident, but there are no results on the specific molecular mechanisms by which UMOD affects ENaC, which will be a direction for future research to focus on and expand. Furthermore, WNK kinases are a family of central kinases that regulate the activity of ion channels involved in blood pressure regulation. Mechanistically, WNK kinases regulate the activity of renal cation-chloride transport proteins through the activation of the SGK1-WNK-SPAK/OSR1 phosphorylation cascade (76), and it is not clear whether UMOD stimulates WNK, or stimulates the upstream kinases of the WNK-SPAK/OSR1 axis, such as SGK1 (serum/glucocorticoid-regulated kinase 1) and protein kinase B (also known as AKT), which is not supported by the literature and will be something to explore in the future. ROMK, a voltage-dependent K + channel, increases K + efflux when ROMK activity is enhanced, largely decreasing intracellular K + concentration, at which point cation channels in the epithelial cells of the renal unit (including NKCC2, NCC, and ENaC) increase the uptake of the corresponding cations to compensate for the negative membrane potential resulting from the increased ROMK activity (77–79). In animal experiments, it has been demonstrated that Umod(-/-) mice exhibit a significant accumulation of ROMK in vesicles due to UMOD deletion, which results in delayed or reduced ROMK expression. Furthermore, the co-expression of ROMK with UMOD has been shown to enhance ROMK-mediated K + transporter activity, leading to an enhanced current amplitude. This effect appears to be dependent on the C-terminus of ROMK. Further investigation of the mechanism of the interactions between ROMK and UMOD reveals that UMOD removes the ubiquitylated C-terminus of the targeting ROMK from binding, thereby promoting an increase in the activity and amount of ROMK (80). In addition, tumor necrosis factor-α (TNF-α) has been widely mentioned in various animal experiments and clinical studies, and both protein and mRNA expression of NKCC2 was significantly up-regulated in TAL cells of TNFα-/- mice, suggesting that TNF-α is an endogenous inhibitor of NKCC2 and can affect the bioavailability and function of NKCC2 (81). TNF-α is produced by TAL and downregulates NKCC2 expression in an autocrine manner, reducing NaCl reabsorption at this site. Several studies have shown that UMOD binds several cytokines through its epidermal growth factor (EGF) structural domain, including TNF-α, which has a high affinity for UMOD (65, 82–85). Lesley A et al. went further by finding that in TAL cells, TNF-α stimulation increased the relative levels of UMOD mRNA, resulting in a negative feedback loop in which TNFα-induced reductions in NKCC2 gene expression were counteracted by increased production of cell surface UMOD (54). It can be concluded that UMOD plays a direct role in blood pressure regulation by modulating the effect of TNF-α on NKCC2 expression. Vasopressin plays an important role in water reabsorption by inducing apical expression of the water channel aquaporin-2 (AQP 2) (86), and because vasopressin is a hormone secreted during dehydration or volume deprivation, upregulation of UMOD secretion by vasopressin is considered to be a plausible physiological response. Recently, vasopressin has been shown to increase UMOD secretion by activating PKA (protein kinase A). In addition, it has been shown that UMOD enhances AQP2 phosphorylation and apical transport in mouse collecting duct cells treated with vasopressin analog dDAVP. The uromodulin-induced apicaltrafficking of AQP2 was attenuated via endocytosis inhibitor treatment, suggesting that uromodulin activates AQP2 through the suppression of endocytosis (87). Moreover, NKCC2 phosphorylation and activity are also regulated by the dDAVP-V2R-cAMP-PKA pathway, in which dDAVP promotes salt reabsorption by upregulating NKCC2 (88). However, in Umod(-/-) mice, attenuated phosphorylation of NKCC2 conferred a dDAVP-resistant phenotype for salt reabsorption, suggesting that activation of NKCC2 by urinary regulatory protein-regulated SPAK/OSR1 kinase superimposed on activation of salt regulation by dDAVP ligands (66). These findings suggest that TAL and collecting ducts work synergistically to retain sodium and water by interacting with urinary modiﬁcation proteins, which in turn leads to hypertension.

In parallel to scientific studies such as animal experiments, advances in UMOD genetics have confirmed that UMOD affects blood pressure. Genome-wide association studies (GWAS) have revealed the existence of variants in the UMOD gene encoding uromodulins that are susceptible to renal function, and hypertension (89), and that non-coding UMOD gene variants induce salt-sensitive hypertension and renal injury through increased expression of uromodulins (55). The relevance of urinary regulators and their role in the regulation of sodium homeostasis is consistent with the results of the human GWAS (90). Single nucleotide polymorphisms in the UMOD gene were associated with hypertension, and conversely, variants with lower UMOD levels were associated with a lower risk of hypertension (91). Mendelian randomization (MR) is an emerging research methodology that allows the use of genetic variation (usually single nucleotide polymorphisms, SNPs) as instrumental variables to simulate randomized controlled trials (92). Using data from more than 750,000 people of European ancestry and applying a two-sample Mendelian randomization method, the researchers found that for every 1-SD increase in uUMOD, SBP increased by 0.06 SDmmHg and DBP increased by 0.08 SDmmHg (93). In another study, a two-sample Mendelian randomization of six datasets of over one million people found that SNPs leading to higher sUMOD and uUMOD were causally related to both SBP and DBP (61, 93). In a study of serum uromodulin and its genetic variants about blood pressure and hypertension in Chinese adults, Chinese researchers found that rs12917707 and rs12708631 in the uromodulin gene were significantly associated with longitudinal blood pressure change over 8 years of follow-up and that rs12708631 was significantly associated with the 8-year incidence of hypertension (94). Mendelian randomization studies using genetic variation as instrumental variables have further substantiated the causal relationship between uromodulin levels and blood pressure. However, these studies are subject to heterogeneity and polyvalence due to environmental factors influencing uromodulin levels, including nutritional intake, volume status, acid-base balance, renal function, and medication use.

The current studies allow us to determine that uUMOD promotes ion channel activity on TAL and DCT to cause sodium and water retention, which in turn leads to hypertension. Since uUMOD levels are associated with an increased risk of salt-sensitive hypertension, measurement of uUMOD is important for the diagnosis of hypertension and already has future clinical significance in guiding the use of diuretics, such as furosemide, in patients with sodium-water retention in this type of hypertension. uUMOD has often been used in various cohort studies of uromodulin and hypertension in populations, however, this does not mean that sUMOD is not associated with hypertension, as sUMOD was found to be significantly associated with a reduced risk of hypertension in a cohort study of adolescents with hypertension in Hanzhong City, China (94). In conclusion, there is a close link between uUMOD and hypertension, and the specific mechanism has not yet been fully explained, which still needs to be supported by continuous exploration of scientific research.

### 4.2 Uromodulin and coronary heart disease

The concentration of sUMOD has been widely proven to be closely related to renal function, and can even be used as the basis for CKD staging, and sUMOD has become self-evident as a biological marker of renal disease (95–98). In recent years, sUMOD has also been gradually emerging in the field of coronary heart disease. In the community-based KORA F4 study, sUMOD emerged as an independent biomarker of cardiovascular event-related mortality in men aged 62 years or older, even after adjusting for confounding clinical factors. Furthermore, sUMOD displayed a significant negative association with total and cardiovascular mortality in men (99). Studies by Steubl et al. indicated that higher levels of both sUMOD and uUMOD were linked not only to a reduced risk of end-stage renal disease (ESKD) in the elderly (100), but also to a decreased risk of cardiovascular disease, including myocardial infarction, stroke, and coronary artery disease or stroke-induced mortality (101). Delgado et al. investigated the association of sUMOD concentrations with cardiovascular biomarkers and mortality risk in a large cohort referred for coronary angiography, revealing that higher sUMOD levels were associated with favorable metabolic profiles, reduced prevalence of comorbidities, and a lower 10-year mortality risk (102). In addition among among white patients with chronic kidney disease, elevated sUMOD levels were associated with decreased risks of mortality, cardiovascular events, and kidney failure (103). Although most of the current studies on sUMOD and coronary heart disease are clinical observational studies, with few animal experiments and other specific elucidations of the mechanism, there are clues to explore the specific mechanisms involved. Atherosclerosis forms the pathogenetic basis of CHD, characterized by endothelial injury, inflammation, and endothelial cell apoptosis (104). Coronary artery calcification (CAC) typically accompanies advanced atherosclerosis development and serves as a predictor of future cardiovascular events (105). Uromodulin has been shown to be an inhibitor of calcification in blood and urine, reducing the risk of calcification (25, 106). Inflammatory pathway activation and oxidative stress drive vascular smooth muscle calcification (107–109), which uromodulin counteracts by interfering with pro-inflammatory cytokine signaling (TNF-a and IL-1b mediated osteochondral signaling) and reducing phosphate-induced calcification in aortic smooth muscle cells (110). Prospective studies demonstrate that higher baseline sUMOD levels predict reduced odds of CAC progression and diabetic kidney disease (DKD) in adults with type 1 diabetes over 12 years (111). While preliminary evidence suggests uromodulin's potential in reducing CHD risk by inhibiting coronary artery calcification, robust support from extensive animal experiments and clinical studies is lacking and warrants further exploration by researchers. Studies suggest a negative correlation between uromodulins and triglycerides (TG) alongside a positive correlation with high-density lipoprotein cholesterol (HDL-C), indicating their potential in modulating lipid metabolism and reducing CHD risk (112, 113). However, the renal disease may influence this relationship, as severe renal impairment may attenuate the protective effects of uromodulin on calcification and lipid metabolism.

sUMOD appears to have a protective effect on the vascular system by inhibiting vascular calcification, so does uUMOD have no role in this? In the Systolic Blood Pressure Intervention Trial (SPRINT), uUMOD concentration twice the norm was correlated with reduced cardiovascular disease risk in patients with chronic kidney disease (114). Sharma's research indicated reduced levels of uUMOD protein in coronary artery disease (CAD), particularly in individuals with recent myocardial infarction, suggesting its potential as an early diagnostic biomarker (115). However, conflicting evidence suggests that uUMOD may not be associated with risks of end-stage renal disease, cardiovascular disease, or heart failure (116), and baseline uUMOD levels and changes therein may not correlate with mortality (117). The precise relationship between uromodulin and cardiovascular disease is subject to the ongoing investigation and may be influenced by factors such as ethnicity, age, and gender. Genome-wide association studies (GWAS) have shown that common variants in the promoter of the UMOD gene with reduced uUMOD levels result in a reduced risk of CVD in the general population (91). A recent Mendelian randomization study demonstrated an effect of higher uUMOD on elevated blood pressure, which mediates the effect on the risk of myocardial infarction in the general population (118). Genetic and experimental findings were not in complete agreement or even had opposite results, suggesting that further studies are still needed to validate the association between uromodulin and coronary heart disease outcomes.

## 5 Future and outlook

While significant strides have been made in understanding uromodulin's role in cardiovascular disease, numerous unknown aspects warrant further exploration. Current research primarily focuses on hypertension and coronary artery disease, leaving other cardiac conditions such as atrial fibrillation, valvular disease, cardiomyopathy, myocarditis, and heart failure relatively understudied. Given the interconnected nature of cardiac diseases and their profound impact on overall health, investigating the relationship between uromodulin and these conditions holds promise for advancing our understanding of cardiovascular pathophysiology. Recent findings suggest an association between uromodulin and left atrial remodeling, as well as left atrium size (119), with implications for conditions like atrial fibrillation, stroke, heart failure, and increased cardiovascular disease rates and mortality (120–124). This area presents an exciting avenue for future research into uromodulin's role in cardiovascular disease. Patients with chronic kidney disease (CKD) face elevated cardiovascular risk, with a significant proportion experiencing cardiovascular events (125). Continued exploration of uromodulin's involvement in cardiovascular diseases holds potential benefits for this patient population, given the high prevalence of cardiovascular complications in CKD. Furthermore, the association of uromodulin, exclusively produced in the kidney, and the UMOD gene, exclusively expressed in the kidney, with cardiovascular disease, opens avenues for investigating cardiorenal syndrome and elucidating the intricate link between heart and kidney health.

## 6 Conclusion

Uromodulins have garnered significant interest among researchers and clinicians in recent years. Progress has been made in understanding their structural characteristics, physiological functions, and clinical relevance. Importantly, their investigation is no longer confined to renal disease, unveiling their enigmatic properties across various body systems. As research advances, uromodulins' distinctive role in the cardiovascular system and beyond is becoming evident. However, further studies are essential to solidify their potential utility as novel biomarkers for cardiovascular disease. Continued exploration holds promise for unlocking the full scope of uromodulins’ impact on human health. Looking ahead, collaborative efforts are needed to explore new avenues of research, such as investigating the mechanisms underlying uromodulins' effects on cardiovascular health and conducting large-scale clinical trials to validate their utility as biomarkers. By doing so, we can harness the diagnostic and therapeutic potential of uromodulin to improve patient outcomes in cardiovascular medicine and beyond. Moreover, considering the multifaceted nature of cardiovascular diseases, future investigations should explore uromodulins' interactions with other biomolecules and their potential modulation of diverse pathological processes. Additionally, elucidating the regulatory pathways governing uromodulins' expression and activity could provide valuable insights into novel therapeutic targets for cardiovascular conditions. Overall, sustained research efforts are crucial to fully comprehend the intricate roles of uromodulins in cardiovascular health and disease. By advancing our understanding in this field, we can pave the way for innovative diagnostic and therapeutic strategies that offer improved outcomes and better quality of life for patients worldwide.
